# Prolactin-Producing Pituitary Carcinoma, Hypopituitarism, and Graves’ Disease—Report of a Challenging Case and Literature Review

**DOI:** 10.3389/fendo.2018.00312

**Published:** 2018-06-06

**Authors:** Rita Bettencourt-Silva, Josué Pereira, Sandra Belo, Daniela Magalhães, Joana Queirós, Davide Carvalho

**Affiliations:** ^1^Department of Endocrinology, Diabetes and Metabolism, Centro Hospitalar São João, Porto, Portugal; ^2^Faculty of Medicine, University of Porto, Porto, Portugal; ^3^Instituto de Investigação e Inovação em Saúde (i3S), University of Porto, Porto, Portugal; ^4^Department of Neurosurgery, Centro Hospitalar São João, Porto, Portugal

**Keywords:** pituitary carcinoma, prolactinoma, Graves’ disease, hypopituitarism, adrenal insufficiency, pituitary surgery

## Abstract

**Introduction:**

The diagnosis of pituitary carcinoma is very rare, requires the evidence of metastatic disease, and has a poor overall survival. Malignant prolactinoma frequently requires dopamine agonist therapy, pituitary surgery, radiotherapy, and even chemotherapy.

**Case description:**

A 19-year-old female presented with galactorrhea, primary amenorrhea, and left hemianopsia. Complementary study detected hyperprolactinemia and a pituitary macroadenoma with cavernous sinus invasion and suprasellar growth. She was treated with cabergoline and bromocriptine without clinical or analytical improvement. Resection of the pituitary lesion was programmed and a non-contiguous lesion of the nasal mucosa was detected during the approach. This metastasis led to the diagnosis of prolactin-producing pituitary carcinoma. After partial resection, the patient was submitted to radiotherapy for residual disease with persistent symptoms. She developed growth hormone deficiency, central hypothyroidism, hypogonadism, and permanent diabetes insipidus. Six years later she was admitted for the suspicion of secondary adrenal insufficiency and thyrotoxicosis. Physical findings, laboratory data, thyroid ultrasound, and scintigraphy achieved the diagnosis of Graves’ disease and hypocortisolism. She was treated with hydrocortisone and methimazole, but central hypothyroidism recurred after antithyroid drug withdrawal. Nine years after the diagnosis of a pituitary carcinoma, she maintains treatment with bromocriptine, has a locally stable disease, with no metastases.

**Conclusion:**

This report highlights an unusual presentation of a prolactin-producing pituitary carcinoma in a young female. The patient had multiple hormone deficiencies due to a pituitary lesion and treatments. The posterior development of hyperthyroidism and adrenal insufficiency brought an additional difficulty to the approach.

## Introduction

Prolactinomas are the most common functioning pituitary tumors. They are generally benign tumors with a favorable prognosis and symptoms are mostly related to hyperprolactinemia and rarely to tumor mass effect (1). However, certain pituitary tumors may exhibit an aggressive behavior invading surrounding structures and can cause life-threatening complications (2). Its management is a challenge for health care professionals and should be performed by a multidisciplinary team. Pituitary carcinoma is rare and the diagnosis requires the evidence of metastatic disease (1–5). Indeed, exhibiting metastatic spread is the unique criterion to diagnose a pituitary carcinoma.

Differentiating atypical from malignant pituitary tumors can be very difficult because metastases at initial presentation are very uncommon (3). Currently, there are no reliable markers or tumor-specific features safely predicting the potential malignancy of a prolactinoma. Increased Ki-67 labeling index (LI), mitotic activity, overexpression of the oncoprotein p53, and genetic background have been studied (3, 6–8). Treatment of pituitary carcinoma commonly requires medical management, pituitary surgery, radiotherapy, and even chemotherapy (1–4). Patients with pituitary carcinoma have an expected poor overall survival (9).

Hypopituitarism with multiple hormone deficits (including central hypothyroidism) can occur due to primary pituitary lesions or just after surgical resection or radiation therapy (3, 10, 11). Hyperthyroidism has a prevalence of 0.75% in Europe and Graves’ disease (GD) is the most common cause (12). Central hypothyroidism and primary hyperthyroidism can coexist. Health care professionals require a high index of suspicion to make the correct diagnosis and treatment of several concomitant hormone disorders.

This case report is an unusual prolactin-secreting pituitary tumor. The patient presented a rare pituitary carcinoma and was treated with multiple therapies. We also describe and discuss the challenge of managing GD in the background of central hypothyroidism, complicated by the concomitant development of central adrenal insufficiency.

## Case Description

A 19-year-old female presented with primary amenorrhea, unintentional weight loss (10 kg in 6 months), bilateral galactorrhea for 4 months, headache, and left hemianopsia in the previous month. Her past medical history was significant for Henoch–Schönlein purpura and obesity (body mass index of 32 kg/m^2^). There was no usual medication or family history of endocrine or autoimmune diseases. At an endocrinology consultation in 2008, physical examination highlighted bilateral spontaneous galactorrhea and left homonymous hemianopsia (confirmed with visual fields). Vital signs and remaining physical and neurological examination were normal.

**Figure 1:**
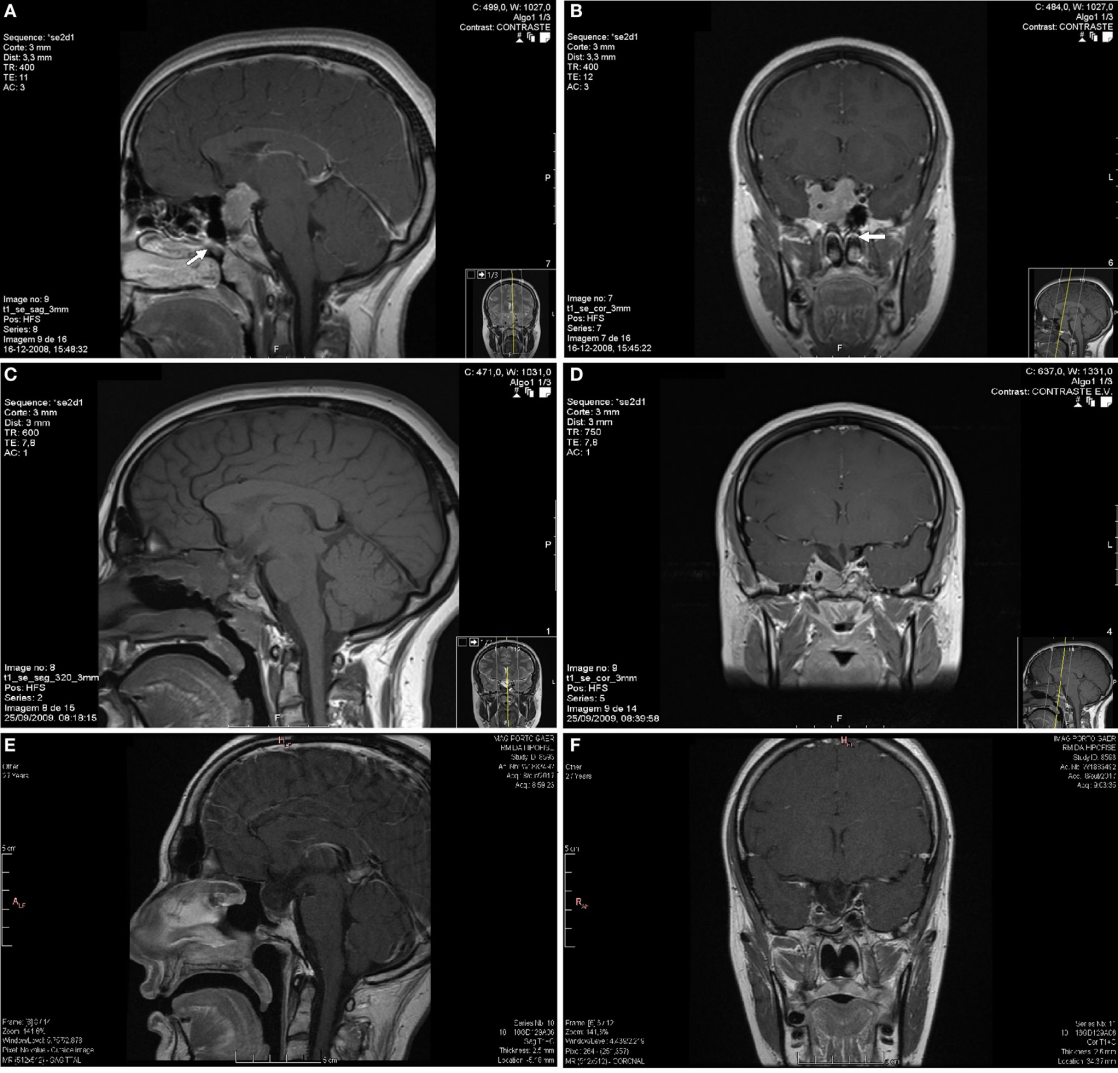
Sagittal **(A)** and coronal **(B)** plane of the first pituitary magnetic resonance imaging (MRI) with an invasive macroadenoma with gadolinium enhancement. The pituitary tumor measured 32 mm × 30 mm × 25.5 mm in anteroposterior, craniocaudal, and transverse dimensions, respectively. The lesion had invasion of the right cavernous sinus, superior deviation of the left internal carotid artery, sphenoid sinus invasion, and compression and stretching of the optical chiasm. The tumor was isossignal area in T1 and T2 with small cysts and had marked contrast enhancement. The possible nasal metastasis was localized near the sphenoid rostrum in the left nasal fossa (arrows). The postoperative MRI **(C,D)** revealed a persistent expansive pituitary lesion with right cavernous sinus invasion. The last pituitary MRI **(E,F)** performed in October 2017 showed a pituitary tumor with markedly reduced dimensions.

Complementary study detected hyperprolactinemia persistently above 200 ng/mL (reference: 1.2–29.9). The samples were not diluted, so it was impossible to obtain the absolute value of prolactin. Renal failure, hypothyroidism, and drug-induced hyperprolactinemia were excluded. Endocrine assessment revealed concomitant growth hormone deficiency and hypogonadotropic hypogonadism. A normal response to insulin-induced hypoglycemia (basal cortisol of 18.0 µg/dL and peak serum cortisol of 26.3 µg/dL after a hypoglycemia of 33 mg/dL) excluded secondary adrenal insufficiency. She underwent a pituitary magnetic resonance imaging (MRI) that revealed a pituitary macroadenoma with right cavernous sinus invasion, sphenoid sinus invasion, and suprasellar growth (Figures 1A,B). The diagnosis of macroprolactinoma was made.

**Figure 2:**
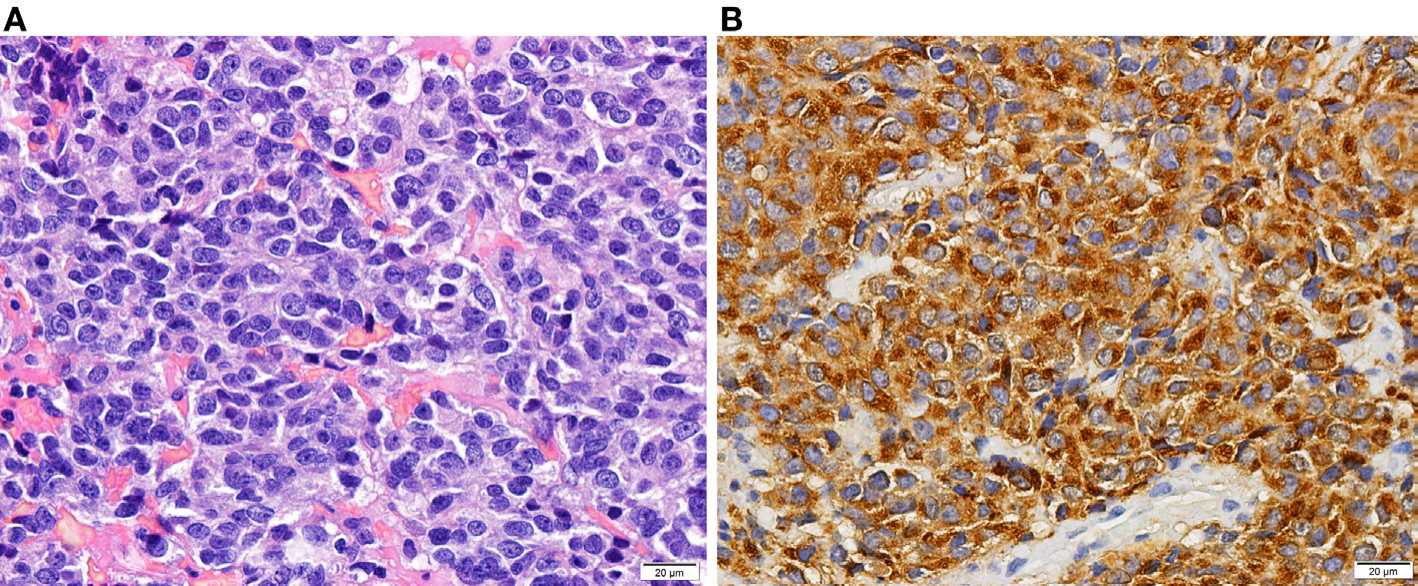
Histology of a prolactin-producing pituitary tumor. Tumor with diffuse growth pattern of cells with elongated nuclei and inconspicuous nucleoli and moderate amount of slightly acidophilic cytoplasm [**(A)**—HE 400×]. Prolactin expression in neoplastic cells [**(B)**—400×].

She was treated for 8 months with two dopamine agonists at the maximum tolerated doses (cabergoline 5 mg/week and bromocriptine 20 mg/day). The combined treatment was made because the patient had side effects with higher doses of bromocriptine, monotherapy with cabergoline was too expensive for the patient and in an attempt to avoid high doses of cabergoline in order to prevent its adverse cardiac effects (in 2008, the safety of cabergoline regarding valvular heart disease in patients with prolactinomas was not guaranteed). However, she had no clinical, analytical, or imaging improvement and was considered resistant to dopamine agonists. The patient underwent transsphenoidal resection of the pituitary lesion in 2009, but complete removal was not feasible. During the surgical approach, an independent mucosa lesion located in the left nasal fossa, near the sphenoid rostrum, was detected and removed. After reassessing the MRI images performed 5 months before the surgery, a millimetric area of difficult visualization was identified in the left nasal fossa that could correspond to the excised nasal metastasis (Figures 1A,B). Histology of the surgical specimen (Figure 2) revealed an adenoma with prolactin expression, diffuse growth pattern, and Ki-67 LI of 4%. The resected nasal lesion also had prolactin expression, confirming the diagnosis of tumor metastasis. Despite the relatively low Ki-67 LI, the detection of a nasal metastasis non-continuous with the pituitary gland was consistent with a pituitary carcinoma. After surgery, she maintained hypogonadotropic hypogonadism and developed permanent central diabetes insipidus and central hypothyroidism with thyroid-stimulating hormone (TSH) levels of 0.06 μUI/mL (reference: 0.35–5.0) and free thyroxine (FT4) levels of 0.76 ng/dL (reference: 0.88–1.58). Desmopressin and levothyroxine were promptly initiated. Estradiol and progestogen were initiated 2 years later due to loss of libido and dyspareunia with significant reduction in quality of life and sexual satisfaction. Serum prolactin levels and tumor size did not change after this treatment.

The postoperative pituitary MRI revealed a persistent expansive lesion with cystic transformation and necrotic areas (Figures 1C,D). The whole-body bone scintigraphy with technetium-99m hydroxymethane diphosphonate showed no focal bone pathology. She also underwent cervical-thoracic-abdominal-pelvic computed tomography, with no additional lesions disclosed. Six months after surgery, she underwent radiation therapy (1.8 Gy/day, 45 Gy in total) for residual expansive lesion and persistent hyperprolactinemia. The lesion became markedly reduced and normalization of hyperprolactinemia was achieved 3 years after radiation therapy, leading to cabergoline withdrawal and treatment only with bromocriptine (bromocriptine was preferred for economic reasons).

In 2015, 6 years after pituitary surgery and radiotherapy, the patient presented with nausea, asthenia, muscle weakness, palpitations, tremor, and unintentional weight loss (15 kg in 2 months). She was admitted for the suspicion of adrenal crisis and thyrotoxicosis. The patient denied excessive intake of levothyroxine; indeed, she self withdrew it weeks before admission. Physical examination highlighted a high-frequency tremor of the hands, sinus tachycardia (resting heart rates above 100–110/min), and a thyroid bruit. She presented normal blood pressure, no fever, and no signs of endocrine ophthalmopathy or dermopathy. The patient did not meet the diagnostic criteria for thyroid storm (13).

**Figure 3:**
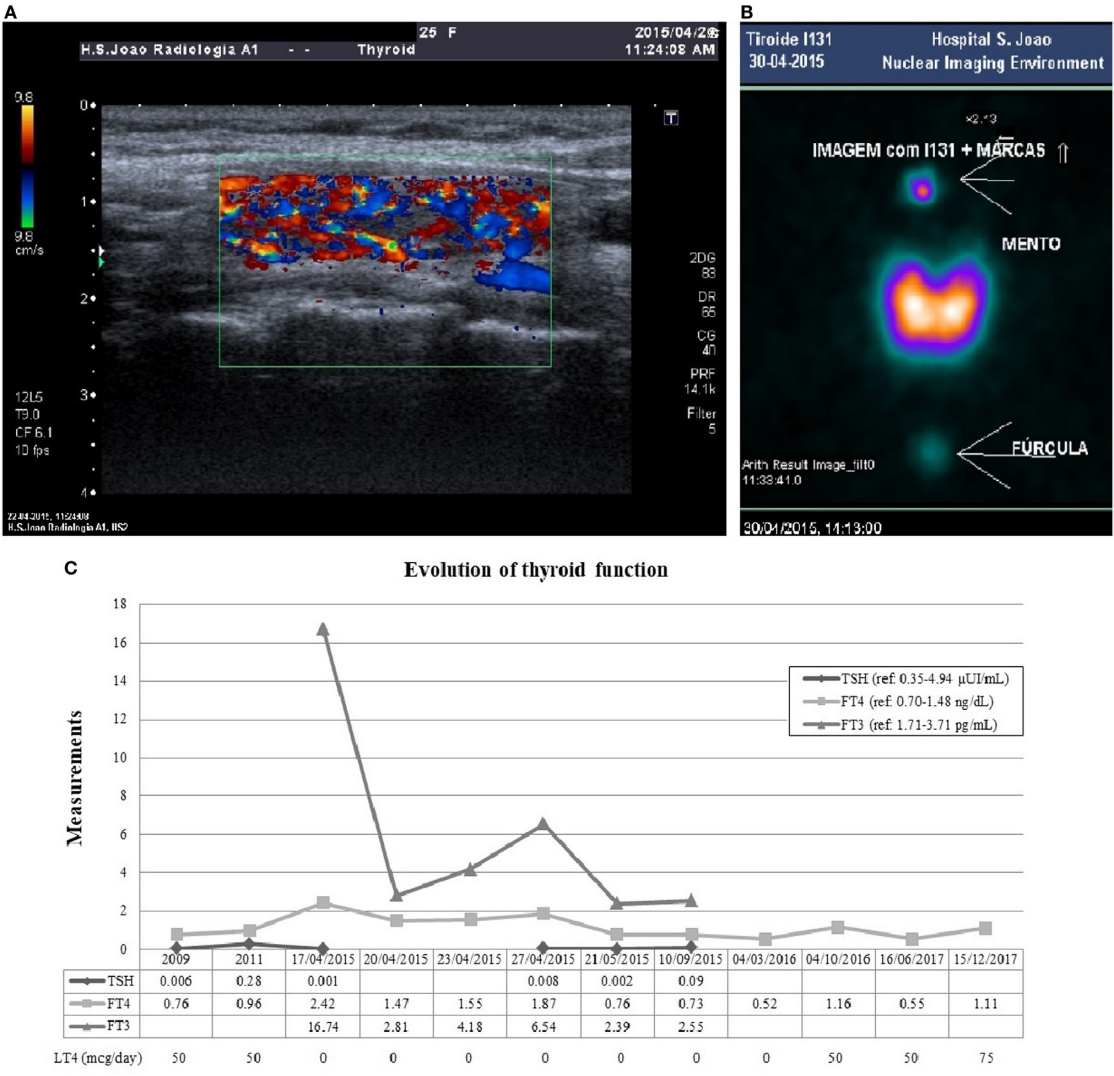
Thyroid ultrasonography **(A)** showed a normal size gland, with heterogeneous texture and pseudonodular areas, without nodular lesions, suggesting thyroiditis. The color flow Doppler signal showed significantly increased vascularity with diffuse homogeneous distribution (thyroid *inferno*). There was a markedly hyperfunctioning thyroid in scintigraphy **(B)**, with homogeneous activity distribution and no focal areas suggestive of hyper- or hypoactive nodular formations. The radioactive iodine uptake was 70.2% at the end of 24 h, markedly elevated compared to normal range (10–30%). Panel **(C)** shows the evolution of thyroid function. After pituitary surgery in 2009 the patient developed secondary hypothyroidism and initiated LT4. She was admitted with primary hyperthyroidism in April 2015 and initiated MMI. During antithyroid drug withdrawal before scintigraphy, FT4 and FT3 re-increased above the reference range. MMI was progressively reduced after 6 months of treatment, but after withdrawal in October 2015, central hypothyroidism recurred and she resumed LT4 since March 2016. Abbreviations: LT4, levothyroxine; MMI, methimazole; FT4, free thyroxine; FT3, free triiodothyronine.

Plasma sampling revealed TSH level of 0.001 μUI/mL (reference: 0.35–4.94), FT4 of 2.42 ng/dL (reference: 0.70–1.48), free triiodothyronine (FT3) of 16.74 pg/mL (reference: 1.71–3.71), and thyroglobulin (Tg) of 7.81 ng/mL (reference: 0–55). Serum FT3 was increased over fourfold while serum FT4 exhibited only a twofold increased, favoring the assumption of primary hyperthyroidism. She had positive thyroglobulin antibody (TgAb) 67.2 IU/mL (reference: <4.11), negative peroxidase antibody (TPOAb) 0.5 IU/mL (reference: <5.61), and TSH-receptor antibody (TRAb) at the upper normal range 1.8 U/L (reference: 0–1.8). Her baseline serum cortisol and adrenocorticotropic hormone (ACTH) levels were low (5.4 µg/dL and 7.2 ng/L, respectively), suggesting secondary adrenal failure. She had mild microcytic anemia and mild leukocytosis, normal renal function, ionogram, and liver profile. Methimazole 20 mg/day, propranolol, intravenous fluids, and hydrocortisone were initiated leading to symptomatic and analytical improvement. To confirm the etiology of thyrotoxicosis, the patient underwent a thyroid ultrasound and scintigraphy (Figure 3). Ultrasonography (Figure 3A) suggested thyroiditis and revealed increased vascularity with diffuse homogeneous distribution. Scintigraphy (Figure 3B) revealed a markedly hyperfunctioning thyroid with a radioactive iodine uptake (RAIU) markedly elevated. These results allowed the diagnosis of GD.

An insulin-induced hypoglycemia test was performed after clinical stabilization to evaluate for adrenal insufficiency. Serum cortisol values below 4 µg/dL secured the diagnosis of long-standing secondary adrenal insufficiency. The patient underwent a progressive methimazole dose reduction and withdrew the drug after 6 months of treatment. At that time, she was medicated with methimazole 5 mg/day and had FT4 levels in the lower normal range. Since she had negative TRAb levels (0.8 U/L) before methimazole withdrawal, the remission was more probable (13). Without any thyroid treatment, she experienced redevelopment of secondary hypothyroidism (low FT4 0.52 ng/dL) and had to resume the treatment with exogenous thyroid hormone. Figure 3C shows the thyroid function evolution since the diagnosis of central hypothyroidism after pituitary surgery.

**Figure 4:**
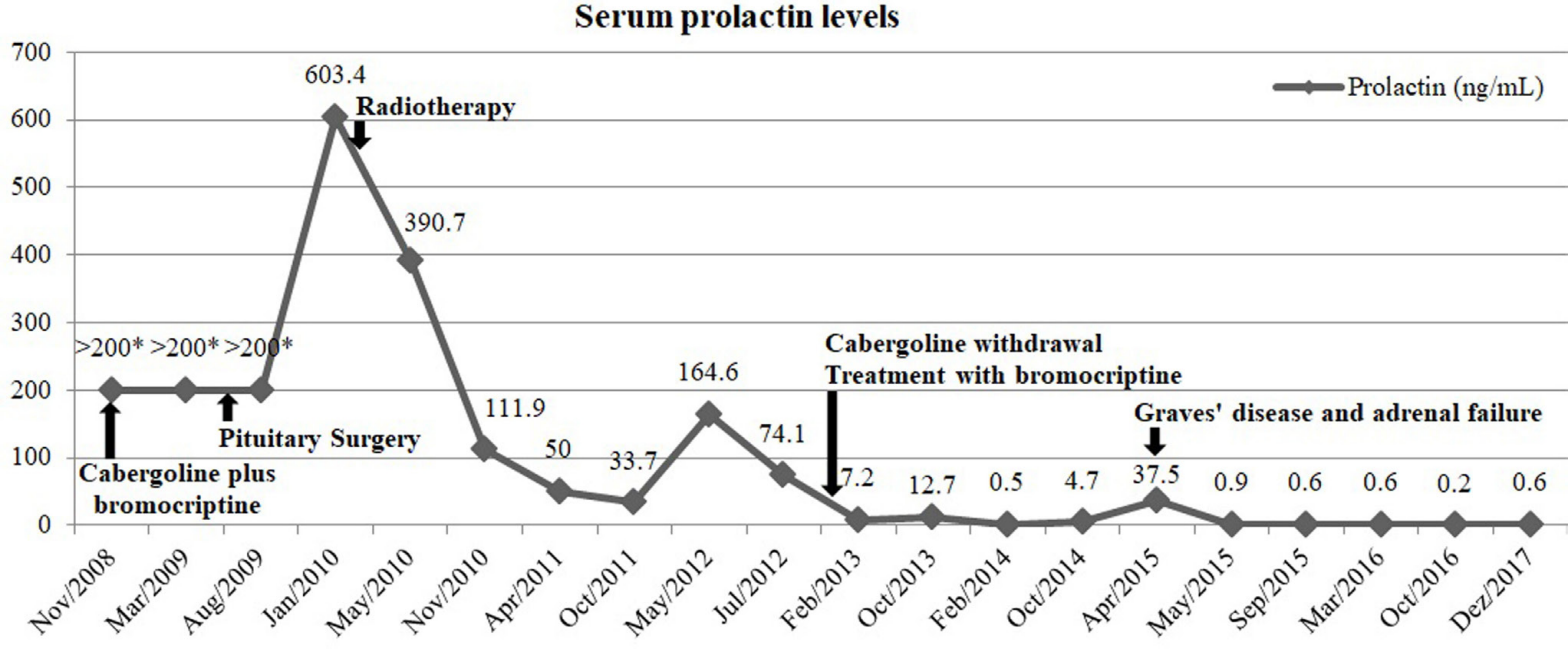
Evolution of serum prolactin levels over time and its relation with medical, surgical, and radiation therapy. *Samples not diluted.

Currently, the patient maintains treatment with levothyroxine and has a normal FT4. She is also under hydrocortisone 20 mg/day, with no symptoms suggestive of adrenal insufficiency or electrolyte disturbances. Concerning the prolactin-secreting pituitary carcinoma, she has normal serum prolactin levels under bromocriptine 20 mg/day and a pituitary lesion with markedly reduced dimensions on MRI compared to pre-radiotherapy period (Figures 1E,F). The evolution of serum prolactin levels since the diagnosis is showed in Figure 4. There was no further evidence of metastatic disease during this 9 years follow-up period.

## Discussion

### Diagnosis of Hyperprolactinemia and Management of Prolactinomas

Prolactinomas are the most frequently secretory pituitary tumors, account for 40% of all pituitary tumors (4). Patients can present signs and symptoms related to tumor mass, like visual field abnormalities, hypopituitarism, headache, and seizures, but also associated with hyperprolactinemia *per se*, like amenorrhea, infertility, and galactorrhea. A single measurement of serum prolactin is enough to confirm hyperprolactinemia and the tumor size usually correlates with serum prolactin levels (1). Patients need to be evaluated for other concomitant hormone hypersecretion or hypopituitarism.

Dopamine agonists are currently the gold standard treatment for both micro and macroprolactinomas (1). Cabergoline has higher efficacy and better tolerability, being preferred to bromocriptine (14, 15). Although most patients present good response to dopamine receptor agonists, in a dose-related manner, others fail to achieve normal prolactin levels and a 50% reduction in tumor size, even at the maximal tolerable doses. Almost 10% of patients are resistant to cabergoline and 25% to bromocriptine (1, 15).

Transsphenoidal pituitary surgery is generally reserved for medically resistant prolactinomas or for patients who cannot tolerate high doses of these drugs (1, 11, 16). A systematic review focused on remission and recurrence of both functioning and non-functioning pituitary adenomas after transsphenoidal surgery (11). Regarding prolactinoma patients, there was a mean remission rate of 68.8% after surgery (similar to those with acromegaly or Cushing’s disease), but a higher recurrence rate was highlighted. A low basal postoperative prolactin and normalization of the thyrotropin releasing hormone test were predictive factors of cure, while age, gender, tumor invasion, and tumor size did not appear to be significant. Surgery-related hypopituitarism developed in less than 10% of patients with prolactinomas (11, 17).

In patients with macroprolactinomas, a careful endocrine follow-up remains necessary if estrogen replacement is initiated. The estrogen receptor is expressed in prolactinomas and estrogens have been implicated in the development and progression of lactotroph tumors, especially during pregnancy (1, 18). However, in patients treated with estrogen/progesterone replacement for 2 years, a rapid growth of an underlying pituitary adenoma was not confirmed (19).

In the case here described, the patient exhibited hyperprolactinemia, growth hormone deficiency, and hypogonadotropic hypogonadism at first endocrine assessment. After surgery, she developed other pituitary hormone deficits, namely thyrotrophic and vasopressin. Interestingly, corticotroph failure was excluded postoperatively with a dynamic test (insulin-induced hypoglycemia test) and was only present 6 years after the pituitary surgery.

### Pituitary Adenomas and Carcinomas

Pituitary adenomas can exhibit aggressive biological behavior and can infiltrate sphenoid sinus, dura mater, and cranial bone (1–5, 8). Pituitary carcinomas are exceedingly rare, making up only 0.2% of all pituitary tumors. Cerebrospinal or systemic metastases are required for diagnosis, but are very uncommon *ab initio* (2, 3, 6). Metastases may be found incidentally or may become symptomatic (3, 20). Patients with pituitary carcinomas have a worse overall survival than those with invasive adenoma (9); once metastases develop, the mean survival is less than 5 years (3).

The World Health Organization classification of Tumors of the Pituitary Gland has been recently revised (21, 22). In the new classification, the previous term “atypical adenoma” is no longer utilized (5). Clinical, biochemical, histological, or tumor-specific radiological features cannot reliably differentiate between locally aggressive adenomas and pituitary tumors (2–6, 8, 20–24). Tumor invasion, higher Ki-67 LI, and elevated mitotic index were correlated with a more aggressive behavior (2, 21). The best marker seems to be the Ki-67 LI, but the best cutoff value varies among studies. In one series (23), malignant tumors exhibited significantly higher mean Ki-67 (11.91%) compared to invasive adenomas (4.66%), suggesting that a Ki-67 LI greater than 10% should raise the concern for malignancy. However, comparing atypical adenomas and pituitary carcinomas identified from the German Registry, there were no significant differences regarding this feature (6). More studies are needed to enable a better identification of increased risk of malignancy, allowing modification of treatment according to aggressiveness, timely management of aggressive tumors, prevention of neoplastic progression, and improvement of outcomes.

Radiotherapy may be required in patients with relevant tumor growth despite surgery and medical treatment in functioning tumors (2). Radiotherapy may require up to 20 years for the maximal effect and is associated with possible hypopituitarism, cranial nerve damage, or second tumor formation (2, 10, 25). Chemotherapy with temozolomide is recommended for pituitary carcinomas after failure of standard therapies (1, 2, 26).

Our patient had a prolactin-producing pituitary carcinoma resistant to cabergoline and bromocriptine. Her nasal metastasis was found and removed during transsphenoidal surgery, but pituitary surgery failed to control disease progression. Radiation therapy led to reduced tumor volume and normal prolactin levels.

### Etiology, Workup, and Differential Diagnosis of GD

Graves’ disease is the most common cause of hyperthyroidism (12). TRAb are GD-specific and an indicator of disease activity (13). Despite being a hallmark in the diagnostic, a significant proportion of patients are TRAb negative. TgAb and TPOAb are detectable in many patients with GD, which tends to develop in a background autoimmune thyroiditis (4). Laboratory and imaging data are crucial in the differential diagnosis of thyrotoxicosis (4, 13). We quickly excluded factitious thyrotoxicosis in our patient because serum Tg would be low or even unmeasurable (4). She had a thyroid bruit and serum FT3 was higher than FT4, favoring the diagnosis of primary hyperthyroidism, because a hyperactive gland produces relatively more T3 than T4 (13). Despite TRAb levels at the upper normal range, TgAb positivity suggested an autoimmune etiology. Thyroid ultrasound was important to confirm the increased vascularity and to exclude nodular lesions. Scintigraphy was helpful to confirm the hyperfunctioning thyroid tissue and to exclude thyroiditis or factitious thyrotoxicosis. Even in the thyrotoxic stage of thyroiditis, RAIU would be low (4, 13); our patient had an unequivocal increased RAIU. Overall, these results led to diagnosis of GD.

Current literature supports the association between hyperprolactinemia and autoimmunity. Prolactin interferes with B and T lymphocyte proliferation and has been associated with autoimmune diseases such as systemic lupus erythematosus, rheumatoid arthritis, psoriasis arthritis, type 1 diabetes mellitus, Addison’s disease, and autoimmune thyroid diseases (27). A recent case-control study found a significantly higher prevalence of autoimmunity, namely thyroid disease, in patients with prolactinomas compared with those with non-functioning adenomas (28). Therefore, chronic hyperprolactinemia may have played a role in triggering autoimmunity in this case.

The most common effect in thyroid function after radiation therapy is overt hypothyroidism, but GD may be a rare radiation-induced disorder (29). The latency time between radiotherapy and thyroid dysfunction is highly variable among studies. In this case report, GD occurred 6 years after radiation therapy. Although unlikely, we cannot exclude the possible relationship between these entities.

### Management of GD in a Patient With Central Hypothyroidism

The presence of central hypothyroidism does not preclude the development of primary hyperthyroidism and these two entities can coexist. In literature, we found some case reports regarding the association between GD and central hypothyroidism. The etiology of central hypothyroidism in the reported cases was craniopharyngioma (30, 31), Sheehan’s syndrome (32, 33), hypothalamic tumor (34), pituitary macroadenoma (35), surgical resection of a pituitary lesion (36), and radiation therapy to a nasopharyngeal tumor with pituitary invasion (37).

The effect of antithyroid drugs in GD is variable among patients. Usually, treatment with methimazole should be continued for 12–18 months and then discontinued when TSH and TRAb levels normalize (13). However, TSH measurement is not helpful in central hypothyroidism, since TSH levels may be low, normal, or even elevated (4). Furthermore, in patients with low or negative TRAb titers, its measurement is less helpful to predict hyperthyroidism relapse after antithyroid drugs withdrawal (13).

### Hyperthyroidism and Adrenal Insufficiency

Thyroid hormones accelerate the cortisol turnover rate by affecting hepatic 11β-hidroxysteroid dehydrogenase type 1 and 5α/5β-reductases (38). Hyperthyroidism or replacement of thyroxine can exacerbate adrenal insufficiency or even precipitate life-threatening adrenal crisis (39). In patients with known hypocortisolism, hydrocortisone replacement therapy may need to be increased in those who develop hyperthyroidism and high dosages of glucocorticoids will acutely block conversion of T4 to T3 (4). On the other hand, physiological concentrations of glucocorticoids exert anti-inflammatory and immunosuppressive actions (40) and hypocortisolism may trigger autoimmunity. Furthermore, some clinical features of both adrenal insufficiency and thyrotoxicosis are similar and a high index of suspicion is required for timely diagnosis.

In stress conditions, ACTH and cortisol levels are expected to increase; a normal cortisol level can be interpreted as relative adrenal insufficiency. A recent study investigated the secretion of cortisol during hyperthyroidism and euthyroidism (41). ACTH-stimulated peak cortisol was decreased during hyperthyroidism state and higher serum thyroid hormones were found to be an independent predictor of this lower cortisol response. In 10% of hyperthyroid patients, the ACTH-stimulated cortisol values were lower than 18 µg/dL (the cutoff generally considered as adrenal insufficiency), but normalized after attainment of euthyroidism. These results alert for the increased possibility of adrenal insufficiency during hyperthyroidism.

## Conclusion

We present the case of a young female with a rare prolactin-producing pituitary carcinoma with a non-continuous nasal metastasis, submitted to medical, surgical, and radiation therapy. The patient had growth hormone deficiency, hypogonadotropic hypogonadism, central diabetes insipidus, and central hypothyroidism related to primary pituitary lesion and treatments. Six years later, she developed adrenal insufficiency and GD. The multiple hypothalamic–pituitary–end organ axis dysfunctions were a challenge in terms of therapeutic approach. To our best knowledge, this prolactin-producing pituitary carcinoma is the only one with a nasal metastasis and has one of the longest survivals after metastases development.

## Ethics Statement

The case report was written with the recommendations of the Declaration of Helsinki. The patient is described anonymously and gave written informed consent for the publication of this case report and any accompanying images. A copy of the written consent is available for review by the Editor-in-Chief of this journal.

## Author Contributions

RB-S participated in the clinical treatment, collected and interpreted the data, and wrote the manuscript. SB, DM, JQ, and DC participated in the clinical treatment and revised the manuscript. JP performed the pituitary surgery, participated in the clinical management, and revised the manuscript. All authors read and approved the final manuscript.

## Conflict of Interest Statement

The authors declare that the research was conducted in the absence of any commercial or financial relationships that could be construed as a potential conflict of interest.

## Abbreviations

ACTH, adrenocorticotropic hormone; FT3, free triiodothyronine; FT4, free thyroxine; GD, Graves’ disease; LI, labeling index; LT4, levothyroxine; MMI, methimazole; MRI, magnetic resonance imaging; RAIU, radioactive iodine uptake; Tg, thyroglobulin; TgAb, thyroglobulin antibody; TPOAb, peroxidase antibody; TRAb, TSH-receptor antibody; TSH, thyroid-stimulating hormone.
